# 聚集相关蛋白基因与转化生长因子β1对肺癌相关生物材料表皮葡萄球菌生物膜形成的影响

**DOI:** 10.3779/j.issn.1009-3419.2014.04.04

**Published:** 2014-04-20

**Authors:** 颖 陈, 玉洁 雷, 云超 黄, 钟鸣 饶, 联华 叶, 光强 赵, 小燕 王

**Affiliations:** 1 650118 昆明，昆明医科大学第三附属医院，云南省肿瘤医院胸外科 Deparment of Thoracic Surgery, the Third Affiliated Hospital of Kunming Medical University, Tumor Hospital of Yunnan Province, Kunming 650118, China; 2 650051 昆明，昆明医科大学附属延安医院心外科 Deparment of Cardiovascular Surgery, the Affiliated Yan'an Hospital of Kunming Medical University, Kunming 650051, China

**Keywords:** 肺肿瘤, 医用硅橡胶, 表皮葡萄球菌, 生物膜, 聚集相关蛋白基因, TGF-β1, Lung neoplasms, Medical grade silicon rubber, Staphylococcus epidermidis, Biofilm, Accumulation associated protein gene, TGF-β1

## Abstract

**背景与目的:**

研究聚集相关蛋白（accumulation-associated protein, *Aap*）基因及转化生长因子β1（TGF-β1）对肺癌相关生物材料表皮葡萄球菌（SE）生物膜形成的影响。

**方法:**

种属鉴定分离临床肺癌患者植入材料感染表皮葡萄球菌株，PCR法检测生物膜形成相关基因*Aap*，检测表皮葡萄球菌*Aap*基因株生物形成能力。密度梯度法提取非小细胞肺癌患者外周血单个核细胞，与人肺腺癌细胞A549在不同浓度（10 ng/mL, 20 ng/mL, 40 ng/mL）TGF-β1共培养30 h后，取上清液分别加入SE Aap+株、SE Aap-株下与医用硅橡胶培养30 h，半定量粘附试验测各组细菌生物膜形成的情况，扫描电镜观察材料表面生物膜微观情况。

**结果:**

*Aap*基因的与表皮葡萄球菌生物膜的形成密切相关（*P* < 0.01）。在（10 ng/mL, 20 ng/mL, 40 ng/mL）TGF-β1浓度组医用硅橡胶表面SE Aap+生物膜的厚度大于空白组（*P* < 0.01）。SE Aap+株在TGF-β1浓度组间生物膜的厚度无明显差异（*P* > 0.05）。在不同浓度TGF-β1因子刺激下SE Aap-株均不能形成明显细菌生物膜。

**结论:**

在肺癌患者植入材料引起感染中表皮葡萄球菌*Aap*基因表达阳性株较易形成细菌生物膜，TGF-β1对SE Aap阳性形成细菌生物膜有一定促进作用。

## Abstract (en)

**Background and objective:**

This study was conducted to evaluate the effects of the accumulation-associated protein (*Aap*) gene and transform growth factor-beta 1 (TGF-β1) on the biofilm formation of lung cancer-related Staphylococcus epidermidis (SE).

**Methods:**

Species identification was performed to isolate SE strains from clinically implanted materials in lung cancer patients. Stable genetic aggregated proteins, which are associated with negative and positive isolates, were obtained. The biofilm-formation ability of the SE Aap gene was determined by PCR. Density gradient method was used to extract peripheral blood mononuclear cells from patients with non-small cell lung cancer. After 30 h, these cells were co-cultured with A549 at different TGF-β1 concentrations. The supernatant was then combined with SE Aap+ and SE Aap- strains and co-cultured with a medical silicone rubber. A semi-quantitative adhesion test was performed for each bacterial biofilm formation. Scanning electron microscopy was also conducted to observe the microcosmic condition of this material on the bacterial biofilm surface.

**Results:**

The *Aap* gene was closely related to SE biofilm formation. At 10 ng/mL, 20 ng/mL, and 40 ng/mL, SE Aap+ biofilm on the medical silicone rubber surface was thicker in the TGF-β1 group than in the control group. No significant differences were found between TGF-β1 groups. For the SE Aap- strains, no evident biofilm was formed in TGF-β1 and control groups.

**Conclusion:**

In plant material-related infection of lung cancer patients, SE Aap+ strain easily forms biofilm. Furthermore, TGF-β1 was conducive for the biofilm formation of SE Aap+ strains.

在肺癌诊疗过程中几乎每位肺癌患者都可能永久或暂时被植入生物材料如：血管导管、外科缝合线、支架、引流管等。肺癌患者自身免疫低下，在接受综合治疗如：手术、化疗、放疗过程中又加重了自身的免疫功能损伤，易并发院内感染，特别是以生物材料为中心的感染（biomaterial centered infection, BCI），是院内感染的高发人群。

课题组前期研究发现由于细菌粘附于植入材料表面形成生物膜（biofilm, BF）以及机体免疫功能失调是造成BCI反复发作，难以控制的关键因素^[1]^。

BCI多由葡萄球菌属引起，特别是定植皮肤、粘膜表面条件致病菌表皮葡萄球菌（staphylococcus epidermidis, SE）常伴随植入过程侵入体内^[2]^。SE粘附于植入材料表面后，其分泌的聚集相关蛋白（accumulation-associated protein, Aap），可促进SE聚集形成BF，是形成细菌生物膜的主要成分。

转换生长因子-β1（transforming growth factor β1, TGF-β1）是恶性细胞分泌的免疫抑制因子之一，是一类功能复杂的细胞因子。由肺癌细胞分泌的TGF-β1不仅与细胞免疫失调密切相关，而且与肺癌的发生、发展以及预后关系密切^[3-5]^。

本实验探讨TGF-β1与表皮葡萄球菌*Aap*基因对肺癌患者医用硅橡胶材料表面生物膜形成的影响。

## 1 材料与方法

### 1.1 试验用菌种及试剂、器材

表皮葡萄球标准株RP62A（生物膜表型阳性，经基因组测序证明存在完整*Aap*基因），ATCC 12228（生物膜表型阴性，经基因组测序证明*Aap*基因缺失）购自中国科学院微生物研究所；人肺腺癌细胞系A549由云南省肿瘤研究所提供；人转化生长因子β1（PeproTech公司，美国）；淋巴细胞分离液（Sigma公司，美国）；API试纸条（BioMerirux公司，法国）；rTaq DNA Marker（Takara公司，日本）；PCR使用所用引物如表 1所示，由大连TaKaRa生物工程有限公司设计，上海生工生物工程有限公司合成。

**1:** PCR引物、复性温度及产物长度 The PCR primers, annealing temperature and the products lengths

Gene	Primers sequences	Product length (bp)	Anealing temperature (℃)
Aap	Aap F 5’-AATGTCCCATACCCTCTA-3’ Aap R 5’-GTT CTCCGT T TATGTCCT-3’	570	58
16s rRNA	16s rRNA F 5’-GGCGACTTTCTGGTCTGTAACT-3’ 16s rRNA R 5’-CTAGAGGGGTCAGAGGATGTCA-3’	285	60
Aap: accumulation-associated protein.			

医用硅橡胶（四川大学生物材料系），将硅橡胶材料加工成面积0.5 cm×1.0 cm大小，甲醛熏蒸24 h灭菌备用。RPMI 1640培养基、DMSO、PBS缓冲液（GIBCO公司，美国）；FBS（COSTAR公司，美国）；电子显微镜EYO LS10（Carl Zeiss公司德国）；紫外分光光度仪、梯度PCR仪（Bio-Rad公司，美国）。

### 1.2 临床表皮葡萄菌的分离、鉴定及细菌DNA的提取

#### 1.2.1 临床表皮葡萄菌的分离、鉴定

留置胸腔引流管的肺癌患者，在排除导管部位发炎或化脓及其他原因的急性发热、寒颤等全身情况下，拔除导管。将导管顶端3 cm浸于肉汤培养基中，经振荡、超声洗脱或离心处理后，使用肉汤进行倍比稀释，种植于血琼脂平板上。根据血琼脂平板上菌落的形态及革兰氏染色，API试剂条行种属鉴定，确认所分离到的细菌为表皮葡萄球菌。

#### 1.2.2 细菌DNA的提取

选取单个葡萄球菌接种于5 mL肉汤培养基37 ℃震荡过夜，加入180 μL的TE缓冲液吹匀，加入20 μL 50 mg/mL溶菌酶溶液37 ℃温育20 min后，加入15 μL的蛋白酶K混匀，55 ℃温育40 min。加入5 μL RNase A震荡样品，静置20 min。加入220 μL BTL缓冲液，混匀后再65 ℃温育10 min。将上清转入新管中，500 μL Buffer HB溶液，10, 000 g离心1 min。1 mL的70%乙醇洗涤后沉淀，离心弃乙醇，重溶于200 μL TE缓冲液中，准备PCR检测。

#### 1.2.3 PCR扩增Aap

DNA、16s rRNA，检测临床表皮葡萄球菌株*Aap*基因以表皮葡萄球菌基因组为模板，16s rRNA为内参，扩增*Aap*基因（表 1）。PCR反应过程：起始变性: 94℃3 min；变性: 94℃30 s；退火（Aap）：58℃30 s，退火（16s rRNA）: 60℃30 s；延伸: 72℃1 min；循环:第2-4步骤35个循环；最终延伸: 72℃10 min。微量加样枪取DNA 2 μL加入的2%琼脂糖凝胶板，加入Marker 2 μL，在电泳槽内进行100 V电泳20 min，成像仪进行检测，检测临床SE分离株Aap基因表达。

### 1.3 表皮葡萄球菌*Aap* 基因在医用硅橡胶上生物膜形成情况

将临床分离SE Aap+株38株，SE Aap-株8株，浓度调整为1×10^6^/mL，与医用硅橡胶在24孔板内培养30 h，取出硅橡胶材料。PBS液进行冲洗3遍，Bouin固定液进行固定1 h，无菌蒸馏水冲洗3遍。使用结晶紫染色3 min，再次无菌蒸馏水冲洗，晾干在微量酶测量标仪上测定490 nm波长吸光度值，测定每株细菌均值。按上述方法测出空白对照490 nm的均值。用实验组值减去空白对照组值，差值> 0.12的菌株即可判断细菌生物膜形成。

### 1.4 非小细胞肺患者PBMCs分离及A549制备

采集非小细胞肺癌患者外周静脉血10 mL，使用淋巴细胞分离液，在2, 000 r/min，温度4 ℃下离心20 min。小心吸出血浆层和淋巴细胞分离液交界的外周血单个核细胞（peripheral blood mononuclear cells, PBMCs）。转移入另一15 mL离心管中加入Hanks液1, 000 r/min离心10 min后洗涤3次，使用含10%PBS的RPMI-1640培养液培养液，置于37 ℃、5%CO_2_培养箱内培养备用。

复苏A549细胞株，用含10%FBS的RPMI 1640培养液，在37 ℃、5%CO_2_培养箱中培养。2天后细胞传代，加入0.25%胰酶消化，取第3代生长旺盛细胞，制备细胞悬液，调整浓度为1×10^6^/mL。

### 1.5 不同浓度TGF-β1下医用硅橡胶表面生物膜的形成

选取SE Aap+株、SE Aap-株（各6株）以及SE标准株，RP62A、ATCC12228浓度为1×10^6^/mL细菌悬液各1 mL。加入在不同浓度（0 ng/mL, 10 ng/mL, 20 ng/mL, 40 ng/mL）的TGF-β1因子下，在浓度1×10^6^/mL的A549细胞，浓度为1×10^6^/mL，PBMCs中共培养30 h上清液中1 mL。后与灭菌硅医用橡胶（每组两片）共培养30 h，半定量粘附试验检测表皮葡萄球菌细菌生物膜形成的情况方法同前。

SEM标本制作和观察用羟乙基哌嗪乙硫磺酸（HEPES）缓冲液（pH=7）冲洗3次，4%戊二醛固定于SEM载物台上，PBS冲洗3遍，二氧化碳（CO_2_）临界干燥，离子溅射表面固定镀膜致PVC材料表面成金黄色，扫描电镜观察细菌生物膜表面结构。

### 1.6 统计学分析

数据使用SPSS 17.0统计软件进行统计学分析，各组的细胞膜生长情况先进行方差齐性检验，若方差齐则采用单因素方差分析进行*F*检验，进一步做两两比较使用*q*检验，*P* < 0.05为差异有统计学意义。

## 2 结果

### 2.1 表皮葡萄球菌临床分离株*Aap*基因PCR检测结果

临床表皮葡萄球菌分离株46株与标准株RP62A、ATCC1228株，经PCR检测：46株表皮葡萄球菌的16s RNA检测阳性率为100%。Aap+株共38株，阳性率82.6%，Aap-株共8株，阴性率17.4%（图 1）。

**1:**
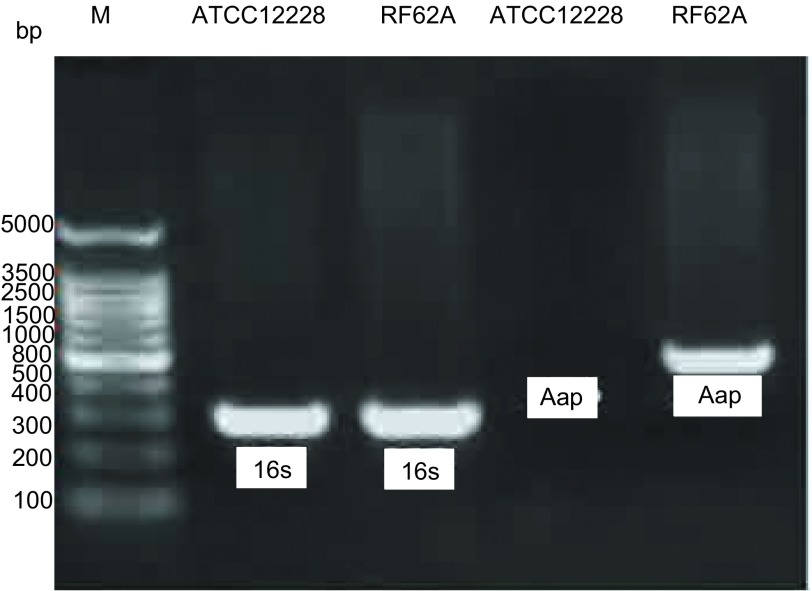
表皮葡萄球菌标准株PCR检测结果 Gel electrophoresis figure of PCR products

### 2.2 表葡菌生物膜的形成能力与*Aap*基因的相关分析

半定量作为判定生物膜形成能力的标准，*Aap*基因的存在与SE生物膜形成密切相关（*P* < 0.01）（表 2）。

**2:** 表皮葡萄球菌*Aap*基因检测阳性率与生物膜表型检测 The Staphylococcus epidermidis *Aap* genetic testing positive rate of biofilm formation detection

Semi-quantitative	*Aap*		Total	Positive rate	*P*
	+	-			
Biofilm (+)	36	0	36	100%	
Biofilm (-)	2	8	10	20%	
Total	38	8	46	82%	< 0.01

### 2.3 TGF-β1因子对SE Aap+株细菌生物膜形成的影响

半定量方法测结果：10 ng/mL、20 ng/mL、40 ng/mL TGF-β1因子刺激下SE986、SE842、SE317、SE276、RP62A细菌生物膜的厚度大于空白对照组（*P* < 0.05）。通过组间比较*q*检验提示20 ng/mL、40 ng/mL组细菌生物膜的厚度无差异（*P* > 0.05）（表 3）。

**3:** 不同浓度TGF-*β*1对A549上清液与外周单个核细胞共同培养下Aap+表葡菌生物膜厚度（Mean±SD） Different concentrations of TGF-*β*1 on the A549 supernatant and peripheral mononuclear cells co-cultured in the Aap positive SE biofilm thickness (Mean±SD)

TGF-*β*1	SE986	SE842	SE505	SE317	SE276	SE945	RP62A
0 ng/mL	0.164±0.002	0.107±0.005	0.151±0.003	0.112±0.003	0.123±0.002	0.135±0.003	0.177±0.005
10 ng/mL	0.192±0.005^Δ^	0.162±0.009^Δ^	0.195±0.007^Δ^	0.224±0.007^Δ^	0.179±0.007^Δ^	0.246±0.006^Δ^	0.225±0.003^Δ^
20 ng/mL	0.331±0.010^Δ^	0.381±0.001^Δ^	0.401±0.003^Δ^	0.407±0.009^Δ^	0.393±0.008^Δ^	0.372±0.003^Δ^	0.355±0.003^Δ^
40 ng/mL	0.481±0.004^Δ^	0.431±0.003^Δ^	0.449±0.008^Δ^	0.446±0.008^Δ^	0.431±0.007^Δ^	0.482±0.001^Δ^	0.472±0.009^Δ^
^Δ^Compared with 0 ng/mL TGF-*β*1 group: *P* < 0.05.							

### 2.4 TGF-β1因子对SE Aap-株细菌生物膜形成的影响

通过半定量方法测量结果：10 ng/mL、20 ng/mL、40 ng/mL TGF-β1因子刺激下的各表皮葡萄球菌的厚度与空白对照组未见差异（*P* > 0.05）。各浓度TGF-β1组间比较未见差异（*P* > 0.05）。实验证明SE Aap-在不同浓度TGF-β1因子刺激（0 ng/mL, 10 ng/mL, 20 ng/mL, 40 ng/mL）均不能形成细菌生物膜（表 4）。

**4:** 不同浓度TGF-*β*1对A549上清液与外周单个核细胞共同培养下Aap-表葡菌生物膜厚度的作用（Mean±SD） Different concentrations of TGF-*β*1 on the A549 supernatant and peripheral mononuclear cells co-cultured in the Aap negative SE biofilm thickness (Mean±SD)

TGF-*β*1	SE709	SE677	SE308	SE965	SE028	SE156	ATCC12228
0 ng/mL	0.123±0.002	0.037±0.005	0.051±0.008	0.102±0.003	0.063±0.002	0.084±0.003	0.027±0.002
10 ng/mL	0.092±0.007^#^	0.057±0.006^#^	0.095±0.008^#^	0.084±0.007^#^	0.089±0.003^#^	0.076±0.005^#^	0.035±0.002^#^
20 ng/mL	0.084±0.007^#^	0.051±0.002^#^	0.071±0.003^#^	0.078±0.010^#^	0.074±0.005^#^	0.069±0.003^#^	0.023±0.002^#^
40 ng/mL	0.072±0.004^#^	0.067±0.004^#^	0.063±0.006^#^	0.076±0.004^#^	0.071±0.002^#^	0.059±0.002^#^	0.043±0.002^#^
^#^Compared with 0 ng/mL TGF-*β*1 group: *P* > 0.05.							

### 2.5 SEM观察不同浓度TGF-β1组医用硅橡胶表面细菌生物膜形成情况

SE Aap+株与RP62A株，在（10 ng/mL, 20 ng/mL, 40 ng/mL）TGF-β1组中，可观察到在医用硅橡胶表面大量SE相互聚集重叠，形成生物膜；在对照组中，仅见少量聚集SE粘附与硅橡胶表面，无生物膜形成（图 2）。

**2:**
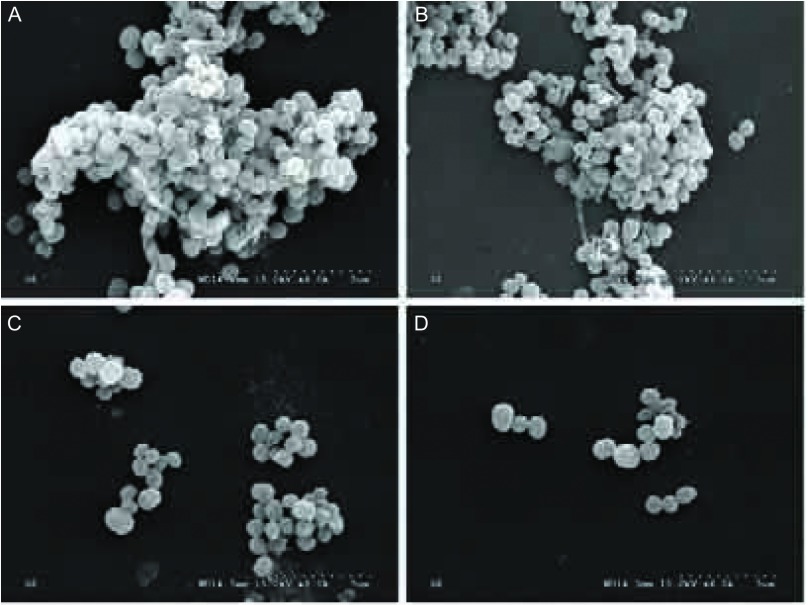
SEM图片。A：40 ng/mL TGF-*β*1组SE Aap+株SEM图片；B: 20 ng/mL TGF-*β*1组SE Aap+株SEM图片；C：10 ng/mL TGF-*β*1组SE Aap+株SEM图片；D：0 ng/mL TGF-*β*1组SE Aap+株SEM图片 SEM pictures. A: The SEM picture of Aap postive SE biofilm in 40 ng/mL TGF-*β*1 gourp; B: The SEM picture of Aap postive SE biofilm in 20 ng/mL TGF-*β*1 gourp; C: The SEM picture of Aap postive SE biofilm in 10 ng/mL TGF-*β*1 gourp; D: The SEM picture of Aap postive SE biofilm in 0 ng/mL TGF-*β*1 gourp

SE Aap-株与标准株ATCC12228株，在（10 ng/mL, 20 ng/mL, 40 ng/mL）TGF-β1组及在对照组中，可观察到在医用硅橡胶表面零散分布单个表皮葡萄球菌，未观察到表皮葡萄球菌出现相互聚集产生细菌生物膜（图 3）。

**3:**
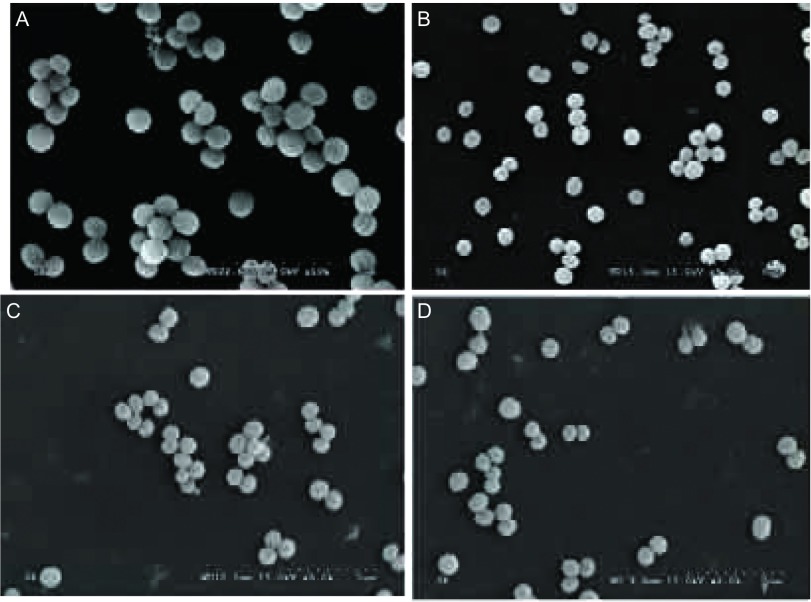
SEM图片。A：40 ng/mL TGF-*β*1组SE Aap-株SEM图片；B：20 ng/mL TGF-*β*1组SE Aap-株SEM图片；C：10 ng/mL TGF-*β*1组SE Aap-株SEM图片；D：0 ng/mL TGF-*β*1组SE Aap-株SEM图片 SEM pictures. A: The SEM picture of Aap negative SE biofilm in 40 ng/mL TGF-*β*1 group; B: The SEM picture of Aap negative SE biofilm in 20 ng/mL TGF-*β*1 group; C: The SEM picture of Aap negative SE biofilm in 10 ng/mL TGF-*β*1 group; D: The SEM picture of Aap negative SE biofilm in 0 ng/mL TGF-*β*1 group

## 3 讨论

研究^[6, 7]^表明，肺癌患者的免疫应答以T细胞免疫为主，肺癌细胞产生的免疫抑制因子TGF-β1是造成癌症患者免疫功能紊乱、Th1/Th2因子失调、T淋巴细胞与NK细胞活性较低的主要原因之一。肺癌患者接受手术、化疗、放疗过程中，更易受条件致病菌感染。

肺癌治疗过程中所植入的生物材料增加了细菌入侵宿主途径，降低了机体的防御能力，BCI上升明显。对于抵抗力较低的癌症患者来说，一旦发生BCI，死亡率高达3%-60%，甚至可达到80%。研究^[8]^表明，BCI与细菌在生物材料表面粘附形成BF密切相关。在TGF-β1因子高表达的肺癌患者，中心静脉导管相关性血流感染几率较高，细菌产生BF的比例也升高。定植皮肤表面的条件致病菌SE是植入材料感染的主要条件致病菌，占肺癌患者BCI的40%。

Aap是一种SE细胞壁锚定蛋白，在BF形成的初期可促进细菌相互粘附，是SE生物膜的主要成分之一^[9]^。Aap结构包括A末端区域和B重复区域。A区域功能在于使SE粘附在人皮肤表面，B区域可以增强其粘附功能。B重复区域由5到17个可变数量的几乎相同128氨基酸组成的保守半重复末端结构，通过Zn^2+^-依赖的D二聚体实现细菌间的直接粘附^[10, 11]^。通过Zn^2+^螯合剂可以抑制SE *Aap*基因的表达，进而影响细菌生物膜的形成。*Aap*基因阴性的表皮葡萄球菌M7株，可以粘附在生物材料表面，但是不能在其表面聚集形成生物膜。

医用硅橡胶价格低廉、易加工、生物相容性良好是临床中最常用生物材料之一，广泛用于制作医用导管、引流管及假体植入等。研究临床分离SE Aap+株与SE Aap-株在医用硅橡胶生物膜的形成。结果提示在肺癌患者医用硅橡胶引流管植入引起感染中，表皮葡萄球菌*Aap*基因与细菌生物膜密切相关，临床分离葡萄球菌Aap-株不能在硅橡胶表面形成细菌生物膜。提示*Aap*基因是表皮葡萄球菌生物膜形成过程中的重要调节基因，调控细菌聚集相关过程，缺乏*Aap*基因所调控的聚集相关蛋白的辅助，表皮葡萄球菌难以在生物材料相互聚集形成细菌群落，最终形成生物膜。

本研究通过分离肺癌患者外周单个核细胞与肺腺癌细胞A549在不同浓度TGF-β1因子刺激下共同培养，模拟体外肺癌免疫抑制状态下表皮葡萄球菌临床分离株在生物材料表面形成过程。结果提示TGF-β1对在肺癌细胞微环境中SE Aap+生物膜形成有一定促进作用。可能是由于肺癌患者T淋巴细胞与NK细胞活性以及体内非特异性杀伤因子水平较健康人群低下^[12]^，TGF-β1的加入进一步抑制肺癌患者PBMCs相关细胞因子的分泌，导致对细菌的杀伤作用下降，细菌更易达到粘附在生物材料表面所需数量。当局部粘附的SE数量达到一定程度后，由于肺癌患者机体因子失调，免疫监视紊乱，可能导致细菌在生物材料上聚集过程不易引发免疫细胞吞噬作用^[13, 14]^。最终导致细菌之间相互粘附、分化、增殖，并分泌大量细胞外粘物质形成BF。

肺癌患者体内免疫因子与TGF-β1作用关系复杂，表皮葡萄球菌细菌生物膜形成的分子学研究不但涉及蛋白依赖，而且还存在多糖依赖等。生物材料植入感染BF形成与生物材料、病原菌、宿主三者的相互关系密不可分。目前国内外对于BCI的研究大多数集中在心血管、口腔、泌尿、整形骨科等学科领域，而对肺癌患者治疗中出现的BCI的研究报道不多，如何分别从生物材料、细菌基因水平、宿主三个方面预防肺癌治疗中BCI值得进一步研究。
